# Health-related quality of life of adults with generalized pustular psoriasis in Malaysia: a cross-sectional study

**DOI:** 10.1186/s13023-025-03820-2

**Published:** 2025-08-27

**Authors:** Xin Qian Seah, Siew Chin Ong, Mustapha Mohammed, Mei Ee Tay, Latha Selvarajah, Wooi Chiang Tan, Sook Yee Michelle Voo, Yen Loo Ting, Jyh Jong Tang, Azura Mohd Affandi

**Affiliations:** 1 Discipline of Social and Administrative Pharmacy, School of Pharmaceutical Sciences, Universiti Sains Malaysia, Pulau Pinang, Malaysia; 2 Biomedical Research Center, QU Health, Qatar University, Doha, Qatar; 3 Department of Dermatology, Hospital Sultanah Aminah, Johor Bahru, Johor Malaysia; 4 Department of Dermatology, Hospital Sultan Ismail, Johor Bahru, Johor Malaysia; 5 Department of Dermatology, Hospital Pulau Pinang, Georgetown, Pulau Pinang Malaysia; 6 Department of Dermatology, Hospital Queen Elizabeth, Kota Kinabalu, Sabah Malaysia; 7 Department of Dermatology, Hospital Kuala Lumpur, Kuala Lumpur, Malaysia; 8 Department of Dermatology, Hospital Raja Permaisuri Bainun, Ipoh, Perak Malaysia

**Keywords:** Pustular psoriasis, Health related quality of life, EQ-5D, DLQI

## Abstract

**Background:**

Generalized Pustular Psoriasis (GPP) is a rare but severe form of psoriasis, characterized by flares involving the sudden spread of erythema with sterile pustules, crusting, and scaling.

**Objective:**

The study aimed to assess both generic and dermatology-specific health-related quality of life (HRQoL) in patients with GPP across flare and non-flare stages in Malaysia.

**Methods:**

The study was a multicenter cross-sectional study conducted among patients diagnosed with GPP attending the General Hospitals under the Ministry of Health, Malaysia, between May 15, 2024, and November 30, 2024. The HRQoL of the GPP patients were assessed using the validated versions of the Dermatology Life Quality Index (DLQI) and EuroQol-5 Dimensions (EQ-5D). Data were statistically analysed using descriptive and inferential statistics using IBM SPSS version 28.0.

**Results:**

A total of fifty-four patients from six centres were enrolled into the study, with a mean (standard deviation, SD) age of 44.6 (SD: 15.5), the majority being female (n = 41, 75.9%). The mean (SD) scores of the DLQI and EQ-5D for the patients with GPP were 8.44 (SD: 7.12) and 0.74 (SD: 0.27), respectively. Thirteen patients (24%) were in their flares phase at the time of assessment. During the flares stage, patients experienced poorer quality of life, as indicated by a significantly higher mean DLQI score of 15.70 (SD: 7.33) (*p* < 0.001), lower EQ-5D score of 0.41 (SD: 0.30) (*p* < 0.001), and an EQ-VAS score of 51.15 (SD: 19.70) (*p* = 0.006), compared to those with non-flares. Patients’ flare status was a significant predictor of impaired HRQoL based on DLQI (*p* = 0.004), EQ-5D (*p* < 0.001) and EQ-VAS (*p* < 0.001) while higher BMI was associated with impaired EQ-VAS score (*p* = 0.001).

**Conclusion:**

The study demonstrates the substantial and dynamic burden of GPP, with a significant impairment in HRQoL among the patients in Malaysia, particularly during disease flares. These findings suggest the importance of prioritizing flares prevention and addressing the holistic needs of GPP patients to maximize their quality of life over the long term.

**Supplementary Information:**

The online version contains supplementary material available at 10.1186/s13023-025-03820-2.

## Background

Generalized Pustular Psoriasis (GPP) constitutes a rare yet severe variant of psoriasis, characterized by extensive pustule formation on the skin, with sudden flares of widespread painful erythema studded with sterile pustules, and often accompanied by potentially life-threatening systemic symptoms [1]. Systemic symptoms of GPP presents with fever, malaise, and joint pain [2].

According to a global Delphi consensus, a GPP flare was defined as the sudden spread of erythema with aseptic pustules, crusts, and scales, accompanied by systemic symptoms like fever, joint pain, and fatigue [3]. During a flare, pustules often merge into large areas and persist for days or weeks [3]. The frequency and severity of GPP flares vary across patients, ranging from multiple flares per year to a flare every few years. In a study conducted in France, most of the patients (79.4%) had one flare while 3% of patients had five or more flares in a year [4]. In a study conducted in Malaysia, the flare frequency of GPP was estimated at 2.2 episodes per year, with a duration of 19.76 days of flare-related admission [5]. GPP flare episodes are a heavy burden to patients, with a significantly longer duration of treatment needed, more inpatient visits, and emergency department visits [6].

According to the 11th Report of the Malaysian Psoriasis Registry (MPR), GPP accounts for about 0.5% of patients with psoriasis and over 60% of those with pustular psoriasis [7]. Data on the prevalence and incidence rate of GPP worldwide remains scarce and varies across geographical regions. In Japan, the estimated prevalence stands at 7.46 per million [8], the prevalence in France was lower, estimated at 1.76 per million patients [9], while the prevalence in Korea was reported higher at 122.3 per million cases [10]. A study conducted in Malaysia noted an increasing trend in prevalence, rising from 178 per million in the year 2010 to 217 per million in the year 2020. This escalation is attributed to higher awareness of this rare disease, potentially indicating a greater economic burden in the future [1].

The debilitating impact of the GPP on patients’ quality of life disrupts daily activities, social interactions, and emotional well-being. Notably, GPP significantly affects patients’ quality of life and exerts substantial economic pressures on the healthcare systems and society [11–14]. Additionally, GPP’s complexity necessitates frequent medical interventions, resulting in substantial costs [11]. Due to the burden of symptoms and the complexity of GPP, understanding the impact of health-related quality of life (HRQoL) can help identify unmet needs and close the gap in patient care. The use of the generic EuroQol-5 Dimensions (EQ-5D) tool is important in estimating the quality-adjusted life years in health economic evaluation studies [15, 16], while the disease-specific HRQoL tool, including the Dermatology Life Quality Index (DLQI) instrument is important from the clinical perspective in improving GPP management [17].

Understanding the patients reported disease burden is significantly challenging due to the rarity, heterogeneity, and relapsing nature of GPP. More data on patient HRQoL in Malaysia needs to be provided. Therefore, this study aimed to assess both generic and dermatology-specific HRQoL in patients with GPP and during flare-ups in Malaysia.

## Methods

### Study design

The study was a multicenter cross-sectional study conducted among patients diagnosed with GPP attending General Hospitals under Ministry of Health (MOH) in Malaysia. The study was guided by the STrengthening the Reporting of OBservational studies in Epidemiology (STROBE) guideline for cross-sectional studies [18].

### Study setting

A total of six study sites were included in the study. The hospitals selected for this study were tertiary hospitals with dermatology services at the Central, Northern, Southern, and East Malaysia. The study sites involved were Hospital Pulau Pinang (HPP), Hospital Raja Permaisuri Bainun (HRPB) from the northern region, Hospital Kuala Lumpur (HKL) located centrally, Hospital Sultan Ismail (HSI) and Hospital Sultanah Aminah (HSA) from the southern region and Hospital Queen Elizabeth (HQE) from East Malaysia.

### Study participants

Adult patients aged 18 years and above, with confirmed diagnosis of GPP, visiting dermatology clinics of MOH general hospitals in Malaysia were recruited from May 15, 2024, to November 30, 2024. GPP was defined as the presence of macroscopically visible, primary, sterile pustules on non-acral skin, not restricted to psoriasis plaques according to the European Rare and Severe Psoriasis Expert Network (ERASPEN) [19].

This study categorized patients with GPP into two distinct group: those experiencing a flare and those in a non-flare state at the time of clinical assessment. The flare period represents the symptomatic phase of GPP, distinguished by the rapid development of erythema covered with aseptic pustules, crusts and scales and associated with systemic symptoms: fever, arthralgia and asthenia [3]. These pustules often merge to big lakes of pustules and can last for days or weeks. Pustular episodes during this stage may persist for days or weeks, and the flaring frequency in GPP is often unpredictable, with episodes occurring several times per year or after long dormant periods. [3]. By contrast, the non-flare period refers to a stage in which patients do not exhibit the hallmark features of a flare. During this phase, there is no development of erythema, aseptic pustules, crusts or scales and systemic symptoms such as fever, arthralgia, and asthenia are absent.

A purposive and convenience sampling strategy was used to ensure a diverse representation of GPP among the study participants. Patients eligible for inclusion were aged 18 years and above with a confirmed diagnosis of GPP by a qualified dermatologist based on the ERASPEN criteria and were referred to the dermatology clinic at General Hospital Malaysia. Patients were excluded if they had defaulted on their regular follow-up appointments in the past year, did not have a scheduled subsequent appointment with the dermatology clinic, or were unable to participate in the interview session.

### Study instruments

The Dermatology Life Quality Index (DLQI) questionnaire was used to assess how skin conditions affect a person’s quality of life of the patients with GPP. It consists of ten questions about various aspects of life impacted by the condition. Respondents choose from response options indicating the extent of impact, which are then scored from 0 to 3. The scores are totaled to obtain a value between 0 and 30, with higher scores indicating a greater impact. A score of 0–1 signifies"no impact"on the patient’s quality of life, while a score of 2–5 suggests a"small impact". Scores between 6 and 10 reflect a"moderate impact", 11–20 indicate a"very large impact", and 21–30 represent an"extremely large impact"[17]. The DLQI assesses the impact of skin conditions on various aspects of a patient’s life, including symptoms and feelings; the ability to engage in daily activities, leisure activities, and work/school; personal relationships; the impact of treatment and medication; the effect on clothing; and the impact on social and recreational activities. The DLQI also includes question assessing emotional well-being and symptom embarrassment.

The EuroQol-5 Dimensions (EQ-5D) questionnaire was used to evaluate patients’ global HRQoL associated with GPP. It measures well-being across five dimensions of health (mobility, self-care, usual activities, pain/discomfort, anxiety/depression), with respondents indicating their health level on a five-point scale. Each dimension has five levels of severity: no problems, slight problems, moderate problems, severe problems, and extreme problems. The questionnaire generates a 5-digit number that represent patient’s health state based on the response from the mentioned five dimensions [15]. The EuroQol Visual Analogue Scale (EQ-VAS) is a self-rated vertical and graduated “thermometer” score (0 to 100 points), with 100 representing “best imaginable health state” and 0 representing “worst imaginable health state”. The utility values for the EQ-5D-5L health states were derived using the Malaysian population-specific value set, based on the MULT8_HYBRID model conducted by Shafie A et al. [20]. For example, a health state of 22345 was computed by applying the weights for MO2, SC2, UA3, PD4, and AD5, and subtracting their total from 1. A lower EQ-5D index represents greater impairment of patients’ HRQoL.

### Study sample size

Given the rarity of GPP, the sample size was determined based on feasibility of patient recruitment rather than a formal power calculation. This approach is consistent with rare disease research, where small cohorts are common due to limited patient availability. The study aimed to describe patients’ HRQoL outcomes (DLQI, EQ-5D, EQ-VAS) in a GPP cohort, with exploratory subgroup analyses. Based on data from the 11th Report of the Malaysian Psoriasis Registry (MPR), which reported 102 GPP patients across Malaysia, a minimum sample size of approximately 30 patients was targeted, reflecting the maximum feasible recruitment within the study period [7].

### Statistical analysis

Data were statistically analysed using descriptive and inferential statistics using IBM SPSS version 28.0. Based on the distribution of data, the continuous variables were reported as means [standard deviation (SD)] or medians [interquartile range (IQR)]. The categorical variables were presented as numbers and percentages. Additionally, EQ-5D and DLQI scores were presented with a mean (SD) and medians (IQR) where appropriate.

Descriptive statistics, including mean, standard deviation, median, and interquartile range, were calculated for patient characteristics, EQ-VAS, EQ-5D and DLQI scores. Inferential statistics of overall EQ-5D-5L and DLQI scores are reported to compare patients with GPP during a flare-up period to those not experiencing a flare-up.

Univariate analyses were conducted to assess the relationship between continuous outcome measures and potential determinants. These analyses included the Mann–Whitney U test and Kruskal–Wallis H test for categorical predictors, and Spearman’s rank correlation coefficients for continuous predictors. Besides, a generalized linear model was conducted to further investigate the factors predicting each outcome measure. Statistical significance was considered at the *p* < 0.05 level at 95% confidence level.

### Ethical approval

The study was registered with the National Medical Research Register and obtained ethical approval from the Medical Research and Ethics Committee (MREC) (NMRR ID-24–00798-OQ3). Informed consent was obtained from all participants before being included in the study. Participants were provided comprehensive information about the study’s objectives, their role in the research, and their right to withdraw without any consequences. Written consent was obtained for both participation and the use of anonymized data in any resulting publications.

## Result

### Sociodemographic and clinical characteristics

A total of fifty-four patients from six centres were enrolled in the study, with the majority being female (n = 41, 75.9%). The mean age of the patients was 44.6 years (SD: 15.5, range: 18–74). Most patients were from the Southern region (n = 26, 48.1%), followed by the Northern region (n = 16, 29.6%) and East Malaysia (n = 8, 14.8%). The mean body mass index (BMI, kg/m^2^) was 27.1 (SD: 6.4), and 27.8% (n = 15) of the population was classified as obese. The average duration of the confirmed GPP was 11.2 years (SD: 9.8). The most common comorbidities were plaque psoriasis (n = 30, 55.6%) and hypertension (n = 15, 27.8%). Among the patients with GPP, thirteen (24%) were in their flares stage during the interview. The detailed patients’ sociodemographic and clinical characteristics are illustrated in Table 1.

**Table 1:** Sociodemographic and clinical characteristics of patients with GPP (n = 54)

Variables	Frequency, n (%)
Age (years), mean (SD)	44.6 (15.45)
*Gender*	
Male	13 (24.1)
Female	41 (76.0)
*State (Region)*	
Southern	26 (48.2)
Northern	16 (29.6)
Eastern	8 (14.8)
Central	3 (5.6)
East coast	1 (1.8)
*Marital status*	
Married	36 (66.7)
Single	15 (27.8)
Divorced	3 (5.6)
*Ethnicity*	
Malay	26 (48.1)
Chinese	19 (35.2)
Indian	3 (5.6)
Others	6 (11.1)
*BMI, (kg/m*^*2*^*)*	
Mean (SD)	27.2 (6.42)
Underweight (< 18.5)	2 (3.7)
Normal weight (18.5–24.9)	19 (35.2)
Overweight (25.0–29.9)	18 (33.3)
Obese (≥ 30.0)	15 (27.8)
*Education level*	
No formal education	2 (3.7)
Primary school	10 (18.5)
Secondary school	24 (44.4)
Tertiary education	18 (33.3)
*Employment status*	
Employed	29 (53.7)
Unemployed	25 (46.3)
*Comorbidities*	
Plaque Psoriasis	30 (55.6)
Diabetes Mellitus	9 (16.7)
Hypertension	15 (27.8)
Dyslipidemia	12 (22.2)
Psoriatic arthritis	14 (26.0)
Bronchial Asthma	3 (5.6)
*Duration of GPP diagnosis (years)*	
Mean (SD)	11.2 (9.81)
< 1	3 (5.6)
1–5	15 (27.8)
6–10	8 (14.8)
> 10	28 (51.9)
*Regular medicine for GPP*	
Topical	36 (66.7)
Oral Systemic	32 (59.2)
Injectable	20 (37.0)
*GPP flares status* Flares	13 (24.0)
Non-flares	41 (76.0)

*GPP* generalized pustular psoriasis; *BMI* body mass index; *SD* standard deviation

### Health-related quality of life scores in patients with GPP

The mean DLQI for the GPP patients (n = 54) in the study was 8.4 (SD: 7.1). The mean EQ-5D and EQ-VAS score for GPP patients in the study were 0.74 (SD: 0.27) and 70.50 (SD: 22.23), respectively. Among the total patients with GPP, those with flares had significantly worse quality of life scores compared to those without flares. The mean DLQI score was significantly higher for patients in their flares compared to non-flares [15.70 (SD 7.33) vs. 6.15 (SD 5.34); *p* < 0.001], the mean EQ-5D score was significantly lower in patients with flares compared to those without flares [0.41 (SD 0.30) vs. 0.85 (SD 0.16); *p* < 0.001)] and also the mean EQ-VAS score was significantly lower in patients with flares compared to those in non-flare periods [51.15 (SD 19.70) vs. 76.63 (SD 19.44); *p* = 0.006]. Details of the comparative analysis of DLQI, EQ-5D and EQ-VAS scores according to flare stages is illustrated in Table 2.

**Table 2:** Comparison of quality of life based on flares status in patients with GPP

HRQoL	Total cohort (n = 54)	Flares (n = 13)	Non-flares (n = 41)	*p*-values
DLQI, mean (SD)	8.44 (7.12)	15.70 (7.33)	6.15 (5.34)	< 0.001*
EQ-5D, mean (SD)	0.74 (0.27)	0.41 (0.30)	0.85 (0.16)	< 0.001*
EQ-VAS, mean (SD)	70.50 (22.23)	51.15 (19.70)	76.63 (19.44)	0.006*

*HRQoL* Health-related quality of life; *DLQI* Dermatology life quality index; *EQ-5D* EuroQol-5 Dimensions; *EQ-VAS* EuroQol visual analogue scale; *GPP* generalized pustular psoriasis; *Statistical significance at p ≤ 0.05 using Mann–Whitney U test

From the results of the DLQI severity assessments, most of the GPP patients (n = 17, 31.2%) experienced a very large impact (DLQI score: 11–20) on their HRQoL, and only three patients (5.6%) reported an extremely large impact (DLQI score: 21–30) on their HRQoL due to GPP. In contrast, 10 patients (18.5%) indicated no impact at all (DLQI score: 0–1) on their HRQoL. Refer to Table 3 for a detailed classification of severity levels based on DLQI score. Across the items in DLQI, items 1 (itchy/sore/painful/stinging skin) was the most affected category. Besides, item 9 (sex life) was the least affected and demonstrated the highest percentages in the"Not relevant"and"Not at all"categories. Refer to Appendix (S1) for further details.

**Table 3:** Quality of life (DLQI severity) based on flare status in patients with GPP

DLQI severity levels	Total cohort (n = 54) n (%)	Flares (n = 13) n (%)	Non-flares (n = 41) n (%)
No effect at all on patient’s life (0–1)	10 (18.5)	1 (7.7)	9 (22.0)
Small effect on patient’s life (2–5)	14 (26.0)	0 (0.0)	14 (34.2)
Moderate effect on patient’s life (6–10)	10 (18.5)	2 (15.4)	8 (19.5)
Very large effect on patient’s life (11–20)	17 (31.5)	7 (53.8)	10 (24.4)
Extremely large effect on patient’s life (21–30)	3 (5.6)	3 (23.1)	0 (0.0)

*DLQI* Dermatology life quality index; *GPP* generalized pustular psoriasis

Among the five domains of EQ-5D, pain/discomfort was the most significantly affected domains, followed by anxiety/depression, with a higher proportion of patients reporting"moderate to severe problems"impacting their HRQoL. In contrast, mobility and self-care were the least affected domains, with more than 50% of patients reporting"no problems"in these areas. Refer to Table 4 for detailed response distribution across EQ-5D domains.

**Table 4:** Quality of life (EQ-5D-5L) based on flare status in patients with GPP

EQ-5D Domains	Total cohort (n = 54) n (%)	Flares (n = 13) n (%)	Non-flares (n = 41) n (%)
*Mobility*			
No problem	30 (55.6)	2 (15.4)	28 (68.3)
Slight problem	10 (18.5)	2 (15.4)	8 (19.5)
Moderate problem	11 (20.4)	6 (46.2)	5 (12.2)
Severe problem	2 (3.7)	2 (15.4)	0 (0)
Extreme problem	1 (1.85)	1 (7.7)	0 (0)
*Self-care*			
No problem	34 (63.0)	3 (23.1)	31 (75.6)
Slight problem	10 (18.5)	1 (7.7)	9 (22.0)
Moderate problem	5 (9.3)	5 (38.5)	2 (0)
Severe problem	4 (7.4)	3 (23.1)	0 (2.4)
Extreme problem	1 (1.9)	1 (7.7)	0 (0)
*Usual activities*			
No problem	27 (50.0)	1 (7.7)	26 (63.4)
Slight problem	15 (27.8)	2 (15.4)	13 (31.7)
Moderate problem	8 (14.5)	6 (46.2)	2 (4.9)
Severe problem	3 (5.6)	3 (23.1)	0 (0)
Extreme problem	1 (1.9)	1 (7.7)	0 (0)
*Pain/discomfort*			
No problem	20 (37.0)	0 (0)	20 (48.8)
Slight problem	16 (29.6)	1 (7.7)	15 (36.6)
Moderate problem	12 (22.2)	7 (53.8)	5 (12.3)
Severe problem	5 (9.3)	4 (30.8)	1 (2.4)
Extreme problem	1 (1.9)	1 (7.7)	0 (0)
*Anxiety/depression*			
No problem	25 (46.3)	3 (23.1)	22 (53.7)
Slight problem	17 (31.5)	3 (23.1)	14 (34.2)
Moderate problem	8 (14.8)	4 (30.8)	4 (9.8)
Severe problem	4 (7.7)	3 (23.1)	1 (2.4)
Extreme problem	0 (0.0)	0 (0)	0 (0)

*EQ-5D*, EuroQol-5 Dimensions; *GPP*, generalized pustular psoriasis

### Predictors of HRQoL in patients with GPP

Univariate analyses were conducted to examine relationships between HRQoL measures and potential determinants. The presence of GPP flares was found to be significantly associated with poorer HRQoL, with higher DLQI scores (*p* < 0.001), lower EQ-5D scores (*p* < 0.001), and lower EQ-VAS scores (*p* = 0.006). Refer to Table 2 for detailed results. Younger age was associated with higher DLQI score (*p* = 0.032). Meanwhile, higher BMI was significantly associated with poorer quality of life, as indicated by higher DLQI (*ρ* = 0.315, *p* = 0.02) and lower EQ-VAS scores (ρ = –0.351, *p* = 0.009,). Additionally, a longer duration of confirmed GPP diagnosis was significantly associated with better HRQoL, as indicated by DLQI (ρ = –0.307, *p* = 0.024) and EQ-VAS scores (ρ = 0.310, *p* = 0.023). Besides, patients receiving injectable therapies reported significantly better HRQoL than those not receiving them, with lower DLQI scores (5.8 vs. 10.0; *p *= 0.030), higher EQ-5D (0.81 vs. 0.70; *p* = 0.012) and EQ-VAS scores (80.2 vs. 64.8; *p* = 0.004). Patients using topical agents had poorer HRQoL, with higher DLQI scores (10.0 vs. 5.4; *p* = 0.041). Patient receiving oral systemic agents showed no significant differences in their HRQoL (DLQI, EQ-5D and EQ-VAS scores). Other predictors including ethnicity, educational level, employment status, and marital status were not significantly associations with DLQI, EQ-5D, or EQ-VAS. Refer to Appendix (S2) for detailed results of the univariate analysis of all predictors.

A generalized linear model was conducted to further confirm the associations of age, BMI, duration of confirmed GPP diagnosis, treatment modalities (topical/injectable) and GPP flare status with various HRQoL measures. The analysis demonstrated that patients experiencing an active GPP flare had poorer HRQoL than those in non-flares, reflected by significantly higher DLQI score (15.70 vs. 6.15; *p* = 0.004), lower EQ-5D (0.41 vs. 0.85; *p* < 0.001) and EQ-VAS score (51.15 vs. 76.63; *p* < 0.001). Moreover, higher BMI was significantly associated with lower EQ-VAS scores. A detail breakdown of the result is illustrated in Table 5.

**Table 5:** Predictors of HRQoL in patients with GPP using generalized linear model

Predictors	DLQI		EQ-5D		EQ-VAS	
	*B* (95% CI)	*p*-value	*B* (95% CI)	*p*-value	*B* (95% CI)	*p*-value
Age (years)	− 0.012 (− 0.024,0.001)	0.08	− 0.005 (− 0.012,0.002)	0.179	− 0.001 (− 0.006,0.004)	0.656
BMI (kg/m^2^)	0.027 (− 0.003,0.057)	0.077	0.007 (− 0.01,0.023)	0.420	− 0.02 (− 0.032, − 0.008)	0.001*
Duration of GPP diagnosis	0.006 (− 0.014,0.025)	0.560	− 0.006 (− 0.018,0.005)	0.264	− 0.001 (− 0.009,.008)	0.888
Treatment modalities Topical Injectable	0.160 (− 0.220,0.539) − 0.211 (− 0.566,0.145)	0.410 0.245	− 0.211 (− 0.429,0.007) − 0.124 (− 0.361,0.114)	0.057 0.307	− 0.075 (− 0.228,0.099) 0.103 (− 0.058,0.263)	0.338 0.210
GPP flares status	− 0.599 (− 1.007, − 0.191)	0.004*	0.782 (0.500, 1.063)	< 0.001*	0.337 (0.150,0.520)	< 0.001*

*HRQoL* Health-related quality of life; *DLQI* Dermatology life quality index; *EQ-5D* EuroQol-5 Dimensions; *EQ-VAS* EuroQol visual analogue scale; *GPP* generalized pustular psoriasis; *BMI* body mass index

^*^Statistical significance at p ≤ 0.05 at 95% confidence interval

## Discussion

This is the first study in Malaysia to assess the HRQoL of patients with GPP with comparisons across different flare status. The study builds on previous studies that reported the lack of detailed information on the flare status and presence of pustules at the time of data collection, leading to potential underestimation of HRQoL impairment during flares and an overestimation of overall HRQoL impairment in chronic GPP [12, 21].

The generic EQ-5D and the disease-specific DLQI are valuable tools for assessing HRQoL in GPP patients. The use of the generic EQ-5D tool is important in estimating the quality-adjusted life years in health economic evaluation studies [15, 16]. In contrast, the disease-specific HRQoL tool, such as the DLQI instrument, is important from the clinical perspective in improving the GPP management [17]. A higher DLQI and lower EQ-5D and EQ-VAS scores indicates a greater impairment of a patient’s HRQoL.

The study found that the mean DLQI, EQ-5D and EQ-VAS scores was 8.44 (SD: 7.12), 0.74 (SD: 0.27) and 70.5 (SD: 22.23) respectively, reflecting a substantial impact of GPP on patients’ HRQoL. The study’s mean DLQI score of 8.44 (SD: 7.12) aligns closely with the US study’s mean DLQI score of 7.8 (SD: 6.8) in GPP population. Additionally, higher EQ-VAS score was observed in this study (70.5, SD: 22.23) compared to the US study (63.4, SD: 23.8) [22]. The differences could be due to variations in the cultural perceptions of health and well-being as well as the GPP severity in these population.

A study conducted in Malaysia in year 2014 reported a mean DLQI score of 12.4 (range 1–28) during patients’ non-flare periods [23]. The DLQI estimated in 2014 was higher compared to the result of this study (2024), which reported a mean DLQI score of 6.15 (range 0–18). This suggests that GPP management has improved over the past decade. This could be due to the deeper understanding of the pathophysiology of GPP and the availability of more advanced technology, such as biologic agents in the country. In recent years, the use of biologic agents such as guselkumab, secukinumab, ustekinumab was proven to improve patients’ HRQoL significantly [24–26].

The overall EQ-5D index for the GPP patient group in our study is lower (mean: 0.74) than the values reported for the general Malaysian population (mean: 0.93) [27]. This finding suggests that GPP patients experienced a greater burden on their overall HRQoL compared to the general population. A study conducted in Sweden similarly reported an impaired EQ-5D score among patients with GPP (mean: 0.613), further supporting the significant negative impact of GPP on HRQoL [21]. While the findings of the Swedish study generally align with our own observations, the slightly lower EQ-5D score reported in Sweden may be attributable to variations in patient demographics, disease severity, or healthcare systems.

GPP is a rare, autoinflammatory, heterogeneity and multisystem sub type of psoriasis, with varied duration and frequency flares up to months [28]. Hence, the understanding of HRQoL during each phase in GPP is crucial for a comprehensive view of the disease’s impact.

During GPP flares, the DLQI score was significantly higher at 15.70, reflecting the overwhelming dermatological burden of acute pustular eruptions, widespread erythema, and associated systemic symptoms such as fever and malaise. This severity is supported in the EQ-5D and EQ-VAS scores of 0.41 (SD: 0.30) and 51.15 (SD: 19.70) which reflects substantial impairments beyond the skin. These findings align with the clinical understanding of GPP flares as acute, debilitating events that disrupt physical function, daily activities, and mental well-being, necessitating aggressive therapeutic intervention to mitigate both cutaneous and systemic effects.

The stage-specific HRQoL patterns observed in this study demonstrates severe impairment during flares mirror trends seen in other dermatological conditions such as plaque psoriasis or atopic dermatitis. However, the rarity and acute presentation of GPP intensify its impact during flares. The persistent effects in the non-flares’ periods highlight the importance of proactive management, while the persistent anxiety/depression in some non-flares patients calls for integrated psychological care. Dermatologists should therefore adopt a multifaceted approach, combining aggressive flare control with long-term strategies to enhance HRQoL across all disease stages.

One key limitation of this study is the absence of a standardized clinical severity score, such as the Generalized Pustular Psoriasis Physician Global Assessment (GPPGA) or Generalized Pustular Psoriasis Area and Severity Index (GPPASI), during data collection. As a result, the associations between clinical severity with the HRQoL could not be studied further. Future studies may consider incorporating validated severity measures such as the GPPGA to allow for a more comprehensive assessment of the relationship between disease activity and patient-reported outcomes.

In univariate analysis, patients on injectable therapies reported better HRQoL compared to those not receiving injectables. However, the injectable therapy group included multiple agents without specific drug names or dosages, limiting attribution of HRQoL benefits to specific therapies. When multivariable models accounted for age, BMI, and flare status, treatment type was no longer a significant predictor of HRQoL, suggesting other factors may play a role. Future studies may use prospective designs, include detailed treatment information, and track HRQoL over time to better inform patient care strategies.

## Conclusions

The study demonstrates the substantial and dynamic burden of GPP, with a significant impairment in HRQoL among the patients in Malaysia, particularly during disease flares. These findings suggest the importance of prioritizing flares prevention and addressing the holistic needs of GPP patients to maximize their quality of life over the long term.

## Supplementary Information

Additional file 1.

## Data Availability

All data generated or analyzed during this study are included in this published article as supplementary information files.
