# Supplementary material for: Health-related quality of life of adults with generalized pustular psoriasis in Malaysia: a cross-sectional study
Source: Orphanet J Rare Dis. 2025 Aug 27;20:460. doi: 10.1186/s13023-025-03820-2 (PMC12392660; doi:10.1186/s13023-025-03820-2)
Supplement: Supplementary file 1 — Additional file 1. [file 13023_2025_3820_MOESM1_ESM.docx]

**Appendices**

S1: Response distribution for DLQI items (n=54)

| **DLQI items (n=54)** | **Not at all**  **n (%)** | **A little**  **n (%)** | **A lot**  **n (%)** | **Very much**  **n (%)** | **Not relevant**  **n (%)** |
| --- | --- | --- | --- | --- | --- |
| 1. Symptoms | 9 (16.7) | 26 (48.2) | 9 (16.7) | 10 (18.5) | 0 (0) |
| 1. Psychological | 21(38.9) | 18 (27. 8) | 14 (25.9) | 4 (7.4) | 0 (0) |
| 1. Daily activities | 27 (50.0) | 8 (14.8) | 9 (16.7) | 8 (14.8) | 2 (3.7) |
| 1. Clothes | 23 (42.6) | 12 (22.2) | 10 (18.5) | 3 (5.6) | 6 (11.1) |
| 1. Social/leisure activities | 22 (40.7) | 12 (22.2) | 10 (18.5) | 6 (11.1) | 4 (7.4) |
| 1. Sports | 17 (31.5) | 15 (27.8) | 5 (9.3) | 9 (16.7) | 8 (14.8) |
| 1. Work/study | 16 (29.6) | 8 (14.8) | 4 (7.4) | 12 (22.2) | 14 (25.9) |
| 1. Relationships | 27 (50.0) | 18 (27.8) | 6 (11.1) | 4 (7.4) | 2 (3.7) |
| 1. Sex life | 21 (38.9) | 5 (9.3) | 2 (3.7) | 3 (5.6) | 23 (42.6) |
| 1. Treatment effects | 16 (29.6) | 19 (35.2) | 12 (22.2) | 6 (11.1) | 1 (1.9) |

S2: Predictors of HRQoL (DLQI, EQ-5D and EQ-VAS)

| **Predictors** | **p-value** | | |
| --- | --- | --- | --- |
|  | **DLQI** | **EQ-5D** | **EQ-VAS** |
| Age | **0.032** | 0.702 | 0.973 |
| Gender | 0.871 | 0.567 | 0.823 |
| State | 0.108 | 0.16 | 0.88 |
| Marital status | 0.847 | 0.322 | 0.329 |
| Ethnicity | 0.669 | 0.607 | 0.656 |
| BMI, (kg/m^2^) | **0.02** | 0.641 | **0.009** |
| Education level | 0.592 | 0.909 | 0.800 |
| Employment status | 0.670 | 0.965 | 0.882 |
| Comorbidities  Plaque Psoriasis  Diabetes Mellitus  Hypertension  Dyslipidemia  Psoriatic arthritis | **0.014**  0.059  0.764  0.794  0.751 | 0.152  0.122  0.748  0.682  0.09 | 0.65  0.226  0.328  0.692  0.175 |
| Duration of confirmed GPP diagnosis | **0.024** | 0.334 | **0.023** |
| Treatment modalities  Topical  Oral Systemics  Injections | **0.041**  0.508  **0.030** | 0.176  0.576  **0.012** | 0.077  0.247  **0.004** |
| Flare status | **<0.001** | **<0.001** | **0.006** |

Univariate analyses included the Mann–Whitney U test and Kruskal–Wallis H test for categorical predictors, and Spearman’s rank correlation coefficients for continuous predictors.

Boldness indicates statistical significance at p <0.05 at 95% confidence interval
